# Salivary Cortisone to Estimate Cortisol Exposure and Sampling Frequency Required Based on Serum Cortisol Measurements

**DOI:** 10.1210/jc.2018-01172

**Published:** 2018-10-03

**Authors:** Robert F Harrison, Miguel Debono, Martin J Whitaker, Brian G Keevil, John Newell-Price, Richard J Ross

**Affiliations:** 1 Faculties of Medicine and Engineering, The University of Sheffield, Sheffield, United Kingdom; 2 Department of Clinical Biochemistry, Manchester University NHS Foundation Trust, Manchester Academic Health Science Centre, The University of Manchester, Manchester, United Kingdom

## Abstract

**Context:**

Population studies frequently measure cortisol as a marker of stress, and excess cortisol is associated with increased mortality. Cortisol has a circadian rhythm, and frequent blood sampling is impractical to assess cortisol exposure. We investigated measuring salivary cortisone and examined the sampling frequency required to determine cortisol exposure.

**Methods:**

Serum and saliva with cortisol and cortisone were measured by liquid chromatography–tandem mass spectrometry in independent cohorts. The relationship between serum cortisol and salivary cortisone was analyzed in cohort 1 using a linear mixed effects model. The resulting fixed effects component was applied to cohort 2. Saliva cannot easily be collected when a patient is sleeping, so we determined the minimum sampling required to estimate cortisol exposure [estimated area under the curve (eAUC)] using 24-hour cortisol profiles (AUC_24_) and calculated the relative error (RE) for eAUC.

**Results:**

More than 90% of variability in salivary cortisone could be accounted for by change in serum cortisol. A single serum cortisol measurement was a poor estimate of AUC_24_, especially in the morning or last thing at night (RE >68%); however, three equally spaced samples gave a median RE of 0% (interquartile range, −15.6% to 15.1%). In patients with adrenal incidentalomas, eAUC based on three serum cortisol samples showed a difference between those with autonomous cortisol secretion and those without (*P* = 0.03).

**Interpretation:**

Accepting that most people sleep 7 to 8 hours, ∼8-hourly salivary cortisone measurements provide a noninvasive method of estimating 24-hour cortisol exposure for population studies.

## Teaser

Accepting that most people sleep 7 to 8 hours, ∼8-hourly salivary cortisone measurements provide a noninvasive method of estimating 24-hour cortisol exposure in population studies.

Measuring cortisol exposure is important in defining health. Even a subtle increase in cortisol exposure may affect health outcomes, and increased cardiovascular risk and mortality are reported in shift workers and in patients with sleep apnea and functioning adrenal incidentalomas (AIs) (1–5). Cortisol deficiency, irrespective of treatment with glucocorticoids, is also associated with elevated mortality rates and poor quality of life (6, 7). In health, serum cortisol demonstrates a distinct circadian rhythm rising from between 2:00 to 4:00 am to peak shortly after waking and decline throughout the day to low levels in the evening with a nadir around 12:00 am (8). Results from a large number of studies from the 1960s to today and using different assays are very consistent regarding this 24-hour rhythm (9). The circadian rhythm of cortisol is altered in shift workers in relation to changes in the sleep-wake cycle, and this results in increased cortisol exposure as judged by the 24-hour area under the curve (AUC) of cortisol (10). The same is true for patients with functioning AIs who have high nocturnal cortisol exposure (11).

The cortisol circadian rhythm has a period of ∼24 hours and can be described mathematically by a Fourier series (cosinor model) (12). Mathematical principles teach us that, in the absence of measurement inaccuracies and other disturbances, the mesor (mean) can be estimated by taking the mean of any number of equi-spaced samples exceeding the total number of harmonically related sinusoidal components (harmonics). Because the mesor is proportional to the AUC of a periodic function (AUC = mesor × period), it provides a means of estimating AUC. However, the cortisol circadian rhythm within individuals has biological variability, and absolute cortisol levels may be determined by other factors, such as genetic sensitivity to glucocorticoids, cortisol production rates, and variations in clearance (13–15), but overall the circadian rhythm of cortisol is similar between populations in different studies (9). Our earlier work suggests that the cortisol rhythm is well modeled by a two-harmonic series (the mesor plus two harmonically related sinusoidal components), therefore suggesting that any three or more equi-spaced samples would lead to a reliable estimate of the mesor, and hence AUC (16). Given the likely presence of random variation, taking a much larger number of equi-spaced samples would be expected to lead to improved estimates by reducing statistical variability; however, the need to minimize the number of samples in clinical trials argues against this.

Cortisol exposure can be estimated by measuring serum, salivary, interstitial, and urine cortisol, and each method has its advantages and disadvantages. The measurement of serum cortisol requires venepuncture, and the stress of venepuncture may itself raise cortisol levels. Urine requires 24-hour collection, which is often incomplete and in all studies shows reduced sensitivity and specificity for diagnosing cortisol excess compared with measurement of serum samples (17). Interstitial measurements require a complex custom sampling apparatus that is not suitable to study large numbers of subjects. Salivary measurement is noninvasive, and samples can be collected with little stress at home or work and are very stable. However, sampling cannot be easily done during sleep. Salivary cortisone is emerging as an improved measure of serum cortisol compared with salivary cortisol because it is derived from serum free cortisol, which is rapidly converted to cortisone in the salivary gland. Salivary cortisone is measurable at low levels of serum cortisol and is not affected by administration of oral hydrocortisone (16, 18, 19).

Many studies have used single measurements of serum or salivary cortisol to make conclusions about cortisol exposure, especially in the field of psychology (20, 21). However, in view of the circadian rhythm of cortisol, these studies are likely to be inaccurate, and there is a need for a more accurate estimate of cortisol exposure. We have previously shown that 94% of the variation in salivary cortisone is predicted by changes in serum cortisol (16). We have now tested this relationship between salivary cortisone and serum cortisol in a population of healthy individuals and in a patient population with AIs, some of whom had autonomous cortisol secretion. We looked at the frequency of sampling required to estimate the AUC of cortisol over 24 hours using serum cortisol and salivary cortisone.

## Patients and Methods

### Healthy volunteer and patient cohorts

Cortisol data from three previously published cohorts of healthy subjects and patients were used for analysis. Cohorts 1 and 2 had measurements of both serum cortisol and salivary cortisone and were used to examine the relationship between serum cortisol and salivary cortisone. All three cohorts had hourly measurement of serum cortisol and were used for analysis of sampling frequency. Meals were not standardized across studies, and none of the female subjects was on estrogen-containing therapy.

- Cohort 1: Fourteen healthy male volunteers with a median age of 28 years [interquartile range (IQR), 25 to 36 years)], weight 83 kg (IQR, 75 to 90 kg), and BMI 25.3 (IQR, 23.1 to 26.3) who had 24-hour hourly sampling for serum cortisol and salivary cortisone from 7:00 am to 10:00 pm measured by liquid chromatography–tandem mass spectrometry (LC-MS/MS) (16).
- Cohort 2: Eight patients with AIs and autonomous cortisol secretion [overnight dexamethasone suppression test serum cortisol >80 nmol/L or 60 to 80 nmol/L with an ACTH <2.2 pmol/L (10 pg/mL), and no features of clinical Cushing disease] and two age-, sex- and BMI-matched groups (six patients with AIs and no excess cortisol secretion and six healthy volunteers). Patients had a median age of 63 years (IQR, 61 to 67 years), weight 73 kg (IQR, 63 to 97 kg), and BMI 28 (IQR, 24 to 33) and had 24-hour hourly sampling of serum cortisol and hourly salivary cortisol/cortisone from 6:00 am to 11:00 pm measured by LC-MS/MS (11).
- Cohort 3: Twenty-eight healthy (nine female) volunteers, mean age 28 years (range, 18 to 56 years), who had undergone 24-hour hourly serum cortisol profiling measured by LC-MS/MS (22).

### Assays

LC-MS/MS analysis for serum and salivary cortisone was performed using a Waters Xevo TQ-MS^TM^ mass spectrometer and a Waters Acquity^TM^ LC system with an electrospray source operated in positive ionization mode (23). The lower limit of quantitation for serum cortisol was 12.5 nmol/L. The interassay imprecision was 8%, 7%, and 6% at concentrations of 80, 480, and 842 nmol/L, respectively. Salivary cortisone was measured with a modified LC-MS/MS assay with lower limits of detection 0.50 nmol/L, intrassay coefficients of variation <7.9%, and interassay <10.3% at 3.6 to 96 nmol/L of salivary cortisone (24).

### Statistical analysis

All statistical analyses were performed using Matlab^TM^ and Microsoft Excel 2010. In cohort 1, linear mixed effects models were used for cosinor and regression analysis to account for intra- and intersubject variability. Model selection was by likelihood ratio test between models, and statistically significant but more complex models with only marginal improvement in either the Akaike or Bayesian Information Criteria were rejected in favor of simplicity. The selected mixed effects model was found to be superior to its fixed-effects equivalent (*P* < 0.001). The random effects component of the mixed effects model was not applicable for use in cohort 2, so only the fixed effects element was retained.

AUC estimation was conducted as follows. AUC_24_ was computed by the trapezium rule. One-sample estimated AUC (eAUC) was computed as 24 times the sampled value. For two-sample eAUC, the earliest start time was selected, and the mean of the corresponding sample and the sample 12 hours later was computed and multiplied by 24. The start point was advanced by 1 hour and repeated until the sample was exhausted. Three-sample eAUC was conducted as described above with samples at baseline, 8 hours, and 16 hours and likewise for four samples.

To account for intersubject variability, we derived the relative error (RE), a measure similar to the coefficient of variation. For each subject we computed the difference between the actual AUC (AUC_24_) and the under-sampled estimates (eAUC) and divided the difference by the AUC_24_, thus removing the intersubject effect.

The sensitivity analysis explored the loss of accuracy (deviation from eAUC) that occurs when samples are not taken at their prescribed times. This was done by taking all possible patterns of sampling 1 hour too early or too late and computing the relative deviation from the “on-time” estimate.

A two-sample Student *t* test with unequal variances was used to examine differences between patients with AIs with and without subclinical hypercortisolism.

### Ethics

All subjects and patients gave full informed consent. For cohort 1, the study received approval from the South East Wales Research Ethics Committee; for cohort 2, the study received approval from East Leeds National Research Ethics Service Committee; and for cohort 3, the study was approved by the South Manchester Local Research Ethics Committee.

## Results

### Relationship between salivary cortisone and serum cortisol

**Figure 1.**
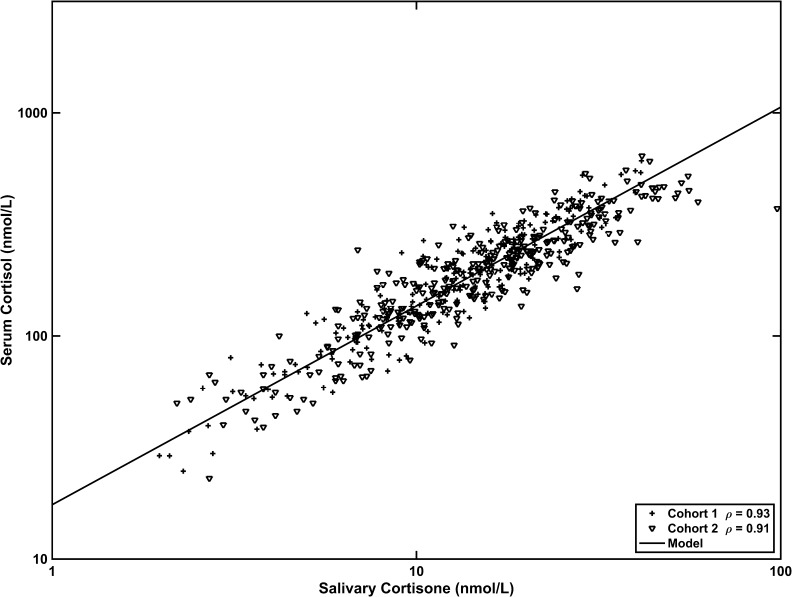
The relationship between serum cortisol and salivary cortisone. Cohort 1 was analyzed using a linear mixed effects model, and the resulting fixed effects component was applied predictively to cohort 2. The relationship is the same in both cohorts.

Application of the fixed effects model (log_10_ serum F = 1.24 + 0.89 log_10_ salE) describing the relationship between serum cortisol and salivary cortisone in cohort 1 was applied to cohort 2, which included patients with AIs with autonomous cortisol secretion as well as matched control subjects. The fixed effects model from cohort 1 gave results similar to those from cohort 2: model predictions of serum cortisol from salivary cortisone gave correlation coefficients of *r* = 0.93 and 0.91 (*P* < 0.001) for cohorts 1 and 2, respectively (Fig. 1).

### Frequency of serum cortisol sampling and comparison of eAUC vs AUC_24_

**Figure 2.**
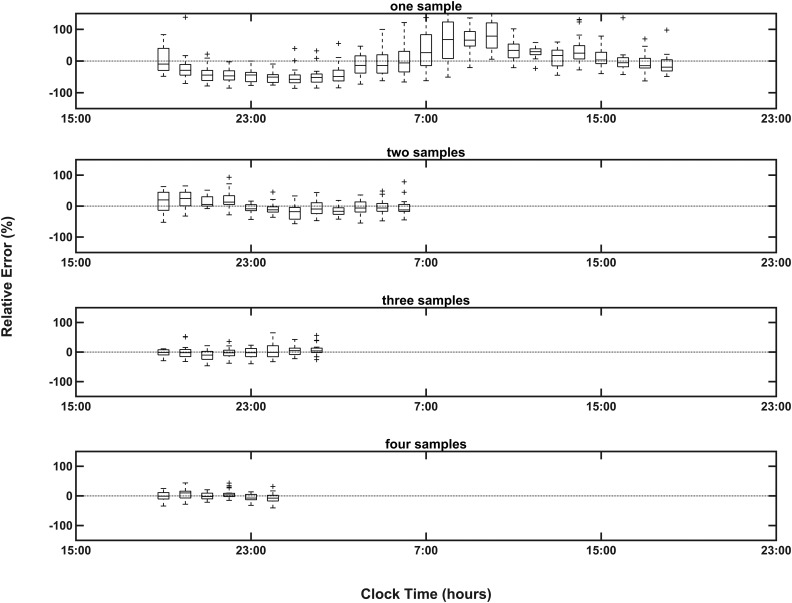
Box-plots of RE with IQR and range across all cohorts using one to four equi-spaced sampling points in estimating AUC. The size of the RE and variation over time decreases with the increasing number of samples measured.

A single sample used to calculate the eAUC was a very poor predictor of the AUC_24_, especially in the morning and last thing at night (Fig. 2). The median RE values were greatest between 7:00 and 9:00 am and between 11:00 pm and 1:00 am (104% to −68%), and the smallest values were between 4:00 and 5:00 am and between 2:00 and 4:00 pm (−42% to 30%). The RE is decreased as two, three, and four equi-spaced samples are used to calculate the eAUC (IQR for the RE with three equi-spaced samples, −15.6% to 15.1%; IQR for four equi-spaced samples, −14.3% to 11.4%). The same pattern was seen when the individual cohorts were analyzed (Table 1).

**Table 1.** RE for Individual Cohorts

	Median	25th Percentile	75th Percentile	IQR
Three equi-spaced samples				
Cohort 1	2.4	−16.5	20.7	37.1
Cohort 2	−1.69	−2.37	1.55	3.92
Cohort 3	−5.78	−11.7	9.39	21.1
All	−0.03	−15.6	15.1	30.6
Four equi-spaced samples				
Cohort 1	2.3	−6.61	6.77	13.4
Cohort 2	−1.53	−7.23	3.37	10.6
Cohort 3	−0.445	−12.1	5.05	17.2
All	−1.11	−14.3	11.4	25.7

### Sensitivity analysis on timing of samples

The 8-hourly sampling scheme is relatively insensitive to mistiming of the samples by up to 1 hour either way for any or all samples. Looking at the variation of the mistimed (±1 hour) three-sample eAUCs against the eAUC on-time across all three cohorts gives a median RE of 0% (IQR, −7.3% to 7.6%).

### Comparison of eAUC vs AUC_24_ in patients with AIs with and without autonomous cortisol secretion

**Figure 3.**
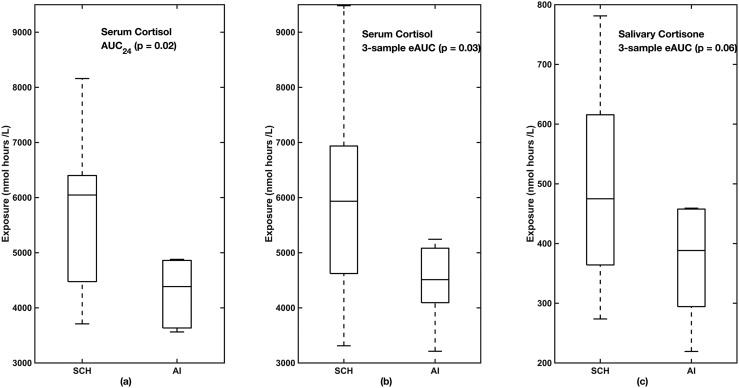
(a) AUC_24_, (b) eAUC for serum cortisol, and (c) eAUC salivary cortisone based on three approximately equi-spaced samples in patients with subclinical hypercortisolism (SCH) and without autonomous cortisol section and AIs. Boxes show IQR. Dotted lines show minimum and maximum range.

To test whether the eAUC could be used to distinguish different patient populations, we examined the AUC_24_ and eAUC between healthy control subjects and patients with AI and autonomous cortisol secretion and those without autonomous cortisol secretion. There was a difference between AUC_24_ for patients with AIs and autonomous cortisol secretion and those without (*P* < 0.02), and the same pattern was seen for eAUC based on three serum cortisol samples (*P* = 0.03). Although the eAUC based on three salivary cortisone samples did not reach significance (*P* = 0.06), the pattern was the same (Fig. 3). The three samples used for serum cortisol were obtained at 7:00 am, 3:00 pm, and 11:00 pm, but, because there was no salivary sample at 7:00 am, the three samples used for salivary cortisone were obtained at 8:00 am, 3:00 pm, and 11:00 pm. The 11:00 pm salivary cortisone in patients with and without hypercortisolemia showed that the 11:00 pm salivary cortisone was higher in the patients with subclinical hypercortisolemia [median (25th to 75th percentiles)]: control subjects, 4.5 (4.0 to 7.9); subclinical hypercortisolism, 9.9 (7.5 to 16.7); and AI, 4.4 (3.0 to 7.4) (*P* = 0.03, ANOVA) with subclinical hypercortisolism different from patients with AIs and control subjects (*P* < 0.05). For the healthy men in cohort 1, the eAUC for salivary cortisone [median (25th to 75th percentiles)] was 406 (387 to 470) nmol h/L, similar to that of the patients with AIs and no autonomous cortisol secretion.

## Discussion

We have confirmed that salivary cortisone provides a good estimate of serum cortisol in populations of healthy subjects and patients. Examining the frequency of serum cortisol sampling, we demonstrate that a single cortisol sample is a poor measure of cortisol AUC, especially when taken around the time of waking or going to sleep. However, three equi-spaced 8-hourly serum cortisol samples give an eAUC with an IQR between −15.6% and 15.1% of the AUC_24_, and this approach was relatively insensitive to mistiming by 1 hour. Taken together, these results suggest that three ∼8-hourly spaced salivary cortisone measurements can give a good estimate of serum cortisol exposure in healthy and patient populations and provide an algorithm for measuring 24-hour cortisol exposure without interrupting sleep independent of the time of starting sampling.

Our data show that a single measurement of cortisol when taken in the morning or last thing at night has a poor correlation with overall 24-hour cortisol AUC. This is in accordance with the problem of AUC estimation from a small number of samples in data that have a periodic component. Estimates can only be unbiased if the number of samples exceeds the number of significant harmonic components needed to represent the curve (*i.e.*, two samples in the case of cortisol). A single sample will always be biased unless its timing matches the point at which the curve crosses the mesor. From our data, the best times for a single measurement in relation to overall cortisol exposure is when the RE is lowest between 2:00 and 4:00 pm or between 4:00 and 5:00 am, corresponding to when the cortisol rhythm crosses the mesor as predicted by theory. Timing of a single sample is tricky in shift workers, whereas taking three ∼8-hourly samples allows sampling to start at any time. The cortisol circadian rhythm is described mathematically by a sinusoid with two harmonics; therefore, three or more equally spaced samples taken over 24 hours should correlate well with the AUC_24_. This is what we observed. Increasing the number of samples will reduce variability in the estimates; however, 6-hourly or more frequent sampling is impractical because it would require sampling during sleep. We found that there was little difference in the accuracy of predicting the AUC_24_ between 8-hourly vs 6-hourly sampling, and even when samples were not taken exactly every 8 hours we found good correlation between the eAUC and AUC_24_.

We are not proposing the salivary cortisone eAUC as a diagnostic test for Cushing syndrome and adrenal insufficiency, where we already have specific and sensitive tests and where cortisol levels at specific times of the day are more relevant than the 24-hour cortisol exposure. The single measurement of either serum or salivary cortisol as a diagnostic test has been used in many studies to investigate Cushing syndrome and disease (17, 25–27). A single late-night cortisol measurement is a sensitive method for diagnosing Cushing syndrome and has been shown to be elevated in some populations such as those with type 2 diabetes (27), and in our study the single measurement of salivary cortisone at 11:00 pm did differentiate functioning from nonfunctioning AIs. However, cortisol exposure (24-hour cortisol AUC) varies in patients with Cushing syndrome and in patients with adrenal insufficiency, and there is overlap between patient populations and healthy individuals. A recent study in patients with Cushing disease showed great variability in late-night salivary cortisol within patients over time (28), and late-night salivary cortisol is a poor marker to differentiate functioning from nonfunctioning AIs (29). We propose that the salivary cortisone eAUC provides an easy-to-administer and more accurate method for comparing cortisol exposure in populations of patients or healthy subjects than single samples or 24-hour serum profiles.

In our small cohort of patients with functioning AIs, excess cortisol secretion would be missed in samples taken in the morning. However, as shown by our data, a sample taken last thing at night or three samples taken ∼8 hourly demonstrated that AIs with excess cortisol secretion, as judged by a dexamethasone suppression test, had overall increased cortisol secretion compared with nonfunctioning AIs. It is likely that adrenal tumors have more stable cortisol excretion, whereas in Cushing disease there may be variability over time. However, taking three samples rather than one is likely to better define the variability related to disease. The salivary cortisone eAUC in the healthy men in cohort 1 was similar to that of patients with nonfunctioning AIs; however, this is not a normal range because a much bigger sample of the population would be required. We know that, in any population of healthy individuals and patients, there is variation in 24-hour cortisol exposure and overlap between patients with excess and deficient cortisol secretion. Therefore, meal times, shift work, and stress can influence cortisol exposure, so in studies comparing populations it is important to control for these factors.

Salivary cortisol has been used as a measurement of free cortisol since the 1960s (30), and now LC-MS/MS provides a highly specific and sensitive method whereby we can measure cortisol and cortisone simultaneously (31). Free serum cortisol is rapidly converted to cortisone in the salivary gland, and salivary cortisone generally shows a better correlation with serum cortisol than salivary cortisol, especially at low levels of serum cortisol where salivary cortisol is undetectable (19). We have previously shown that salivary cortisone reflects serum cortisol using a mixed effects model, and we have now shown that its fixed effects component demonstrates an almost identical relationship in another healthy volunteer population as well as in patients being investigated for AIs, half of whom had functioning adrenal adenomas secreting cortisol. The results confirm that salivary cortisone is a good method for estimating serum cortisol levels, and further studies are required to establish its use. Saliva collection has the advantage of being noninvasive, samples can be collected in a nonclinical setting, and, because steroids are very stable, samples can be posted to the laboratory without any special conditions.

Limitations of our data are the retrospective analysis and that the patient population is relatively small. This is reflected in the fact that the difference in eAUC for salivary cortisone between patients with AIs with or without excess cortisol secretion did not reach significance. However, the studies analyzed provide comprehensive data of hourly sampling over 24 hours in three different subject cohorts, and the results are consistent over the different cohorts. Although this analysis is retrospective, all the studies were done under carefully monitored controlled conditions. Two AUCs can be the same, but the rhythm may be different; it is difficult to define the rhythm from limited sampling, and this will generally require more frequent sampling.

This study provides a strong basis for using three ∼8-hourly spaced salivary cortisone samples when estimating cortisol exposure in healthy and patient populations. This methodology will allow further investigation of the impact of cortisol secretion on health.

## Abbreviations:

AI: adrenal incidentaloma
AUC: area under the curve
AUC_24_: area under the curve for 24-hour cortisol profile
eAUC: estimated area under the curve
IQR: interquartile range
LC-MS/MS: liquid chromatography–tandem mass spectrometry
RE: relative error
